# The consequences of compromising the EU’s free movement of persons principle on Swiss research: how to survive constrained access to regional funding

**DOI:** 10.1186/s12961-021-00684-3

**Published:** 2021-03-06

**Authors:** Jasmina Saric, Miriam Bolz, Marco Waser, Michael Käser

**Affiliations:** 1 Swiss Tropical and Public Health Institute, P.O. Box, CH-4003, Basel, Switzerland; 2 University of Basel, P.O. Box, CH-4003, Basel, Switzerland

**Keywords:** Horizon 2020, Research funding, Switzerland, Free movement, Partial association status, Third country, Collaborative proposals, Collaborative projects

## Abstract

Between 2014 and 2016, Switzerland’s access to some of the EU funding was limited after a referendum against mass immigration was accepted and the country refused to sign the free movement accord to the EU’s newest member, Croatia. It is well documented that Switzerland has suffered from a drop in participation, funding and a decrease in consortium lead positions. However, there is no account of the consequences on institutional level. We therefore aimed at describing the immediate- and longer-term impact of the partial association status to the Swiss Tropical and Public Health Institute (Swiss TPH) and to identify key strategies for minimizing institutional damage during a limited access period to a key regional funding source. A quantitative analysis of the institute’s grants database, from 2007 to 2019, did not show any clear trends related to the partial association status of Switzerland for funding and projects awarded. The qualitative outcomes changed along the timeline assessed; whereas in 2014 a range of negative effects were stated by Swiss TPH researchers, a survey conducted in 2019 with Swiss TPH applicants and project partners to Horizon 2020, revealed that most project leaders felt that the partial association did neither affect their external partners’ willingness to collaborate nor Swiss TPH’s role in the proposal or consortium. On the other hand, the institutional strategic goal of taking on consortia leads was delayed by several years as a direct consequence of the partial association. Also, the exclusion from European research networks and the lack of consultation of expertise by the European partner institutions was widely seen as damaging. A policy of favouring long-term partnerships over ad-hoc collaborations, along with constant and trustful communication, as immediate mitigation measure, helped averting some of the reputational and access damage. Moreover, the Swiss TPH business model based on a three-way strategy of research, education and services has proven highly viable allowing to build a large pool of potential funding sources internationally, resulting in relative resilience in terms of income lost.

## Background

### Switzerland—a scientist Utopia?

Switzerland is an important contributor to global research and innovation. The country, home to a population of 8.5 million, features consistently among the top three countries in Europe as measured by competitiveness, innovation and scientific output [1–4]. Moreover, Switzerland is also the most successful country in securing grants from the European Research Council (ERC) on a per capita basis [3], those grants being widely perceived as a distinction for research excellence. In addition, Switzerland has a central role in some of the most prestigious and important transnational initiatives of modern times including Europe’s particle-physics laboratory [5] and the 10 year Human Brain Project [6]—one of the largest projects the EU has ever funded.

Switzerland owes part of its success to a relatively high research and development (R&D) expenditure as a percentage of the Gross Domestic Product. As per 2017, this was 3.3% [7] compared with the Organisation’s for Economic Cooperation and Development (OECD) average of 2.4%. Its private sector is highly research-oriented and boasts two of the global top-10 companies measured by R&D volume (i.e. Novartis and Roche). In addition, the country seems to have an exceptional pull for international talent attracting professionals from across the globe to research in academia and the private sector. Indeed, more than half of the PhD holders employed at the 12 universities, more than half of all doctoral students and almost half of the countries’ private sector R&D staff are non-Swiss [3].

### The challenges of direct democracy to research and innovation

While enjoying a close-to-perfect base for the pursuit of world-class research most of the times, at least once per decade the Swiss research and innovation community is being shaken by one of the referendums that are a key feature of the Swiss direct democracy. In a crucial mandatory referendum in 1992, the Swiss population rejected joining the European Economic Area leaving EU-Swiss relations somewhat sour ever since [8]. Yet, Switzerland had access to the main EU funding instruments for research and innovation, on the base of a bilateral agreement. In 2005, a 5 year moratorium was imposed on the import and cultivation of genetically modified plants and animals within Switzerland incentivizing some researchers to find more liberal pastures elsewhere or alternative research foci. In February 2014, the largest shock wave to date hit the Swiss research and innovation scene when a referendum against mass immigration was narrowly accepted and, one week later, Switzerland refused to sign the free movement accord for Croatia—the EU’s newest member—offending one of the very core principles of the EU treaty [8, 9]. The European Commission (EC) reacted by suspending all negotiations with Switzerland on bilateral cooperation in research and education for as long as the accord was to remain unsigned [10, 11].

### Swiss association to EU funding

**Fig. 1:**
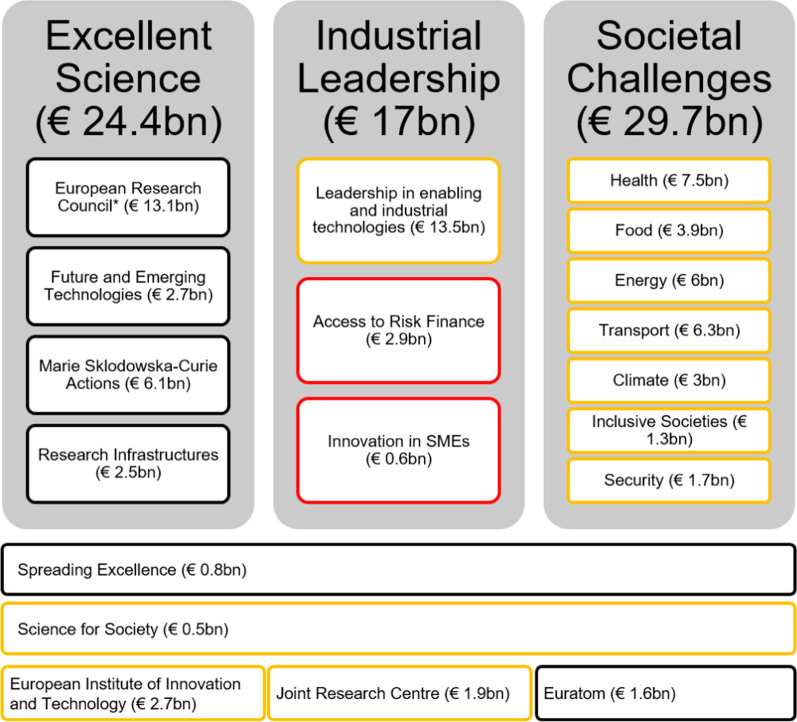
Switzerland’s participation in Horizon 2020 during 2014–2016. *A temporary scheme was put in place by the Swiss Government to support access to the European Research Council (ERC) Schemes; black frame, full association; yellow frame, partial association as third country; red frame, no association during 2014–2016

The EU Framework Programmes for Research and Technological Development (FPs) have been running since 1984 and are now in the 8th phase (named ‘Horizon 2020′). Switzerland, having been involved on a project basis with official third country status initially, received full association status in 2004 enabling the country to participate in parts of FP6 and the full FP7 period [12]. However, in September 2014, as a consequence of the free movement disagreement between the EU and Switzerland, the association status to EU funding schemes of the latter was revised as follows: Switzerland would retain its full association status for all schemes under the first pillar of Horizon 2020 (Excellent Science), including the ERC grants and Marie Skłodowska-Curie Actions (MSCA). For these, successful applicants would still receive funds directly from Brussels, while for all other calls including those of the second and third pillar—Industrial Leadership and Societal Challenges—Swiss participation was downgraded to third country status (Fig. 1) [12]. In practice, this meant that Swiss applicants could still submit project proposals alongside European partners but not in a coordinating position. While evaluation of the Swiss part would still take place in Brussels, the EU would not directly fund it. These new orders followed an 8-month period of uncertainty where the status of Switzerland was not defined at all. In order to maintain a *status quo* for Switzerland-based researchers, the Federal Council arranged for the State Secretariat for Education, Research and Innovation (SERI) to take over the funder’s role as an interim measure, until the eligibility of the Swiss researchers was clarified [13]. Yet, the financial means made available at home were underexploited, as noted retrospectively by a marked decrease in project associations in those areas funded by the Swiss Confederation [14], and they were largely unknown to the non-Swiss research community.

As per January 2017, Switzerland retrieved full association status after having adopted an application law in December 2016 that moved the country back in line with the EU’s bilateral agreements. At the same time, the Swiss Federal Council ratified the protocol extending the free movement of persons to Croatia and therefore fulfilling the necessary condition for Switzerland’s full association to the entire Horizon 2020 programme [15]. Almost two years on, a retrospective analysis of SERI has shown that the participation of Switzerland-based researchers in Horizon 2020 has recovered since 2016. Yet, it has overall weakened as measured by the proportion of Swiss participation with 3.2% in FP 7 (2007–2013), 1.8% in Horizon 2020 (by July 2020) and 2.4% in Horizon 2020 (by March 2018). A similar trend was seen for the proportion of funding received (4.3%, 2.2% and 2.4%) and the proportion of Swiss coordinated projects (3.9%, 0.3% and 2.6%) [15, 16].

The objective of this work was to describe the immediate- and mid-term impact of the temporary partial association status on a Swiss research institution and to identify key strategies for minimizing institutional damage during periods of access limitation to national or regional funding sources.

## Methods

Our research was based on quantitative and qualitative information obtained from the Swiss Tropical and Public Health Institute (Swiss TPH), an intermediate-size research institution with ~ 800 staff, associated with the University of Basel in the north-western part of Switzerland. Swiss TPH is a world-leading institute in global health, tropical medicine and parasitology, hosting research, education and services to enhance the health and wellbeing of populations internationally, nationally and locally. The institute is characterized by an extensive international network of collaborators across the globe. Because of its domain of work and the largest proportion of Swiss TPH operations taking place in low- and middle-income countries (LMICs) outside Europe and because most FP7 and Horizon 2020 instruments were designed to fund societal challenges of Europe, EU funding amounts to 3–5% of the total Swiss TPH budget, while the remaining competitive funding is obtained elsewhere. Yet, funding by the European Commission (EC) has an important role for consortia building, networking and visibility in the global health community.

Data was retrieved from the Project & Grant Service (PGS) Unit’s database that has been recording data relevant to projects and the grant application process at Swiss TPH since 2010. Upon project start, every project leader is obliged to enter comprehensive data of the project into the project database including co-investigators, volume and funding organisation. The project database is the basis for Swiss TPH project reporting to the outside including the annual report. The entry is supervised by each Head of Unit of the institute and curated by a project database responsible. The data made available at Swiss TPH were compared and complemented with information on FP7 and Horizon 2020 awarded projects logged on the CORDIS webpage for EU research results [17]. The CORDIS database is maintained by the EC and was consulted for all FP7 and Horizon 2020 schemes, but not the European & Developing Countries Clinical Trials Partnership (EDCTP) that is supported under Horizon 2020 but widely presented as a self-standing funding scheme.

Quantitative analysis was conducted of all FP7, Horizon 2020 and EDCTP grant application data documented by the PGS Unit to analyse the effects of the partial association on quantity and strategy of funding applications, success-rate and funding for (i) all EU funding schemes including personal fellowships, i.e., the grants from the ERC and MSCA; and (ii) EU funding schemes for collaborative projects (consortia) only.

Qualitative data was extracted from documentations, reports and written and oral communications mostly at the level of the PGS Unit and the directorate of Swiss TPH. In 2014, a first discussion of how the partial association of Switzerland affected Swiss TPH and its capacity to attract Horizon 2020 and EDCTP funding was conducted informally with project leaders at Swiss TPH. In addition, two formal surveys were conducted in 2019: (i) one in-house survey with former applicants of Horizon 2020 grants; and (ii) their non-Swiss collaborating partners in those projects. Questions aimed primarily at the experience made in collaborative proposals and projects to identify challenges at Swiss TPH and when collaborating with Swiss TPH during Switzerland’s partial association status for Horizon 2020 and whether and how those challenges were overcome.

## Outcomes

### Institutional vs national trends in research applications and funding received from the EU in 2007–2019

The documentation of data on grant proposals in-house was deemed reliable and complete from 2007 onwards; therefore, the period 2007–2019 was assessed for all FP7 and Horizon 2020 grant schemes except for EDCTP for which a separate analysis was conducted. The data retrieved on EDCTP was found to be less complete, therefore only projects that took place between 2010 and 2019 were included in the analysis. The project start and end-dates were deemed complete enough to offer a true representation of events.

**Fig. 2:**
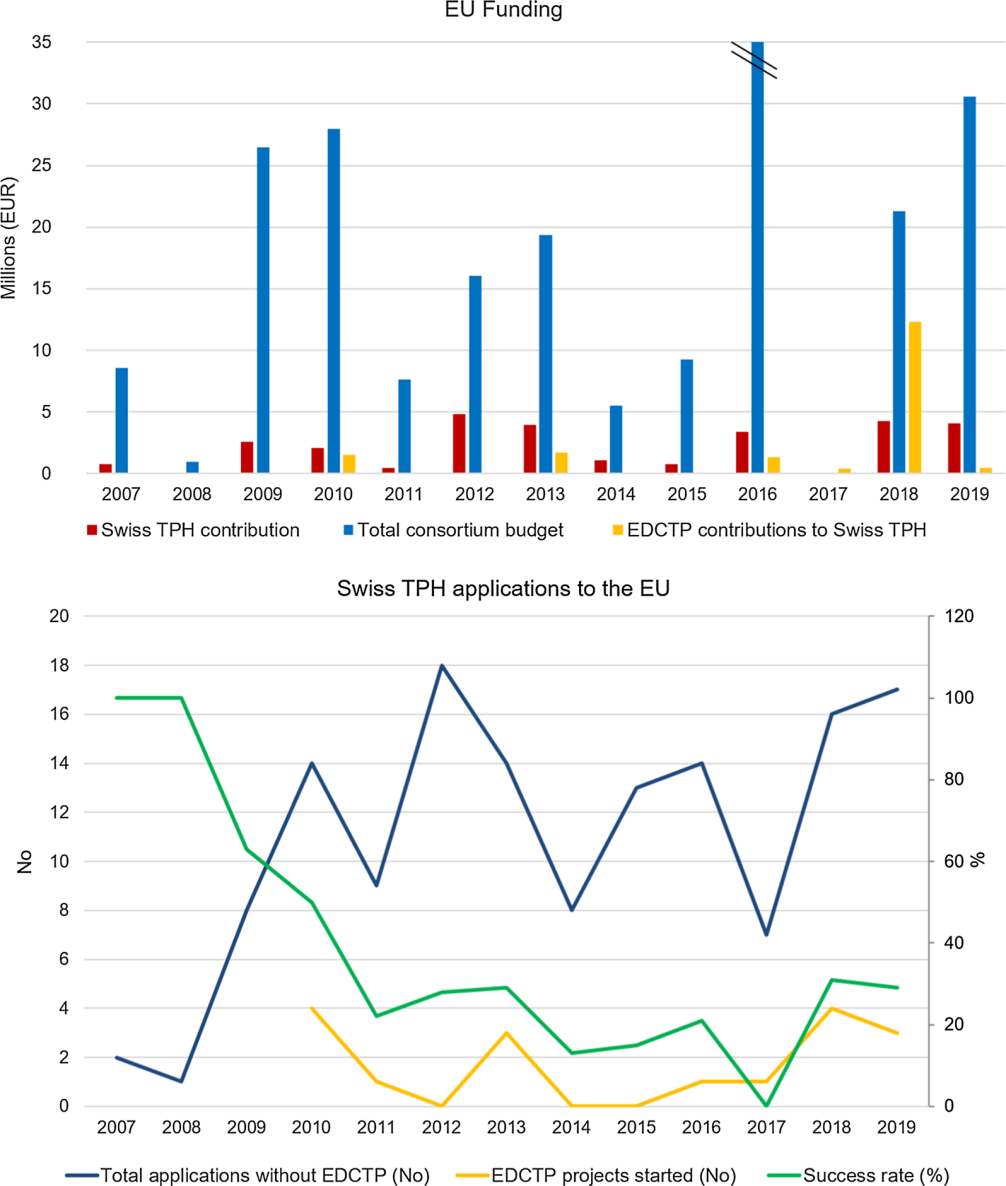
FP7, Horizon 2020, including European Research Council and Marie Skłodowska-Curie Actions, and European and Developing Countries Clinical Trials Partnership (EDCTP) contributions to Swiss TPH and success rates. The large amount of funding received in 2016 relates to the association to the HBM4EU project with a total consortium budget of almost 70 Mio Euro. For EDCTP grants where the award year was more difficult to track back for each proposal, the award year was assumed to equal the year of the project start

The comparison of the funding that Swiss TPH received from those EU schemes between 2007 and 2019, however, did not allow concluding on trends pertaining to the partial association status of Switzerland. The years below average (CHF 2′182′327) were spread across the timeline, i.e., 2007, 2008, 2010, 2011, 2014, 2015 and 2017 (Fig. 2). For the proportion of funded proposals out of submitted proposals, a general downward trend was observed in the period 2007–2017 reaching a minimum in 2017 and increasing thereafter. The total number of proposal applications increased after 2008 showing from 2010 onwards the same pattern of minima and maxima as the curve representing the success rate.

The same analysis for collaborative projects only, excluding personal fellowship applications to the ERC and MSCA, did not change any of the trends observed in the full analysis.

Similar to the FP7 and Horizon 2020 schemes, the projects awarded to Swiss TPH by EDCTP were fluctuating along the time axis (Fig. 2).

There is no doubt that Swiss research as a whole has suffered during the partial association period for Horizon 2020. This has been shown by numbers [15, 16], as well as opinions—including those of the international research community starting to question the value of a Swiss partner and Switzerland as a reliable base for cutting-edge research. However, on the level of Swiss TPH the effects on participation, funding and coordination patterns do not seem to show the same prominent trends as found on national level. Although Swiss TPH does show sub-average levels of successful proposals in some of the years of the partial association (2014 and 2015) as well as in 2017 when potential carry-over effects may have applied, this seems part of a longer trend at Swiss TPH where the proportion of successful proposals has declined between 2007 and 2017, recovering subsequently. The number of total applications seems to be positively linked with the annual success-rate. For the EDCTP grants awarded to Swiss TPH, it was even more difficult to show a clear impact of the partial association phase of Switzerland, based on the limited data available for this single funding scheme.

One factor for the relative resilience of Swiss TPH may be a relatively small reliance on Horizon 2020 funding (3–5% of the total Swiss TPH annual budget of around 90 Mio CHF), with the remaining competitive funding obtained elsewhere. Consequently, relatively minor fluctuation from the average of total annual research funding at Swiss TPH (CHF 25.4 Mio) were observed between 2014 and 2019 (23.7–27.1 Mio). Even the associated scheme—EDCTP—being an important EU instrument to Swiss TPH researchers, is represented by a relatively small average number of starting projects per year (1.4) between 2010 and 2019 at Swiss TPH.

### Switzerland’s partial association to Horizon 2020 and impact perceived at the institutional level

A first stock-taking of how the partial association of Switzerland affected Swiss TPH and its capacity to attract Horizon 2020 and EDCTP funding was conducted informally in 2014 with project leaders at Swiss TPH. This was initiated in response to a Swiss-wide request for information on the consequences of partial association to Horizon 2020 by the Rector’s Conference of Swiss Universities and the Swiss National Science Foundation. The official response of the PGS Unit on behalf of Swiss TPH is summarized in Table 1.

**Table 1:** Consequences of the partial association to Horizon 2020 as experienced by Swiss TPH on the levels of funding, individual researcher and institution

1. Partners cutting ties with Swiss TPH
2. Missed opportunities—Swiss TPH is less frequently approached for collaboration
3. Deprioritizing Swiss TPH contribution—partners need to find an additional partner with first-country status leading to disproportionate large consortia and leaving the Swiss partner in a satellite position rather than as a valued full contributor
4. Uncertainties, rumours and misunderstandings by coordinating partners towards the eligibility of Swiss partners for different European Union instruments
5. More time is invested for (i) communication on the topic of Switzerland’s status as third country; and (ii) proposal development with partners for administrative tasks
6. Challenges to being consortium coordinator leading to loss of institutional credibility and visibility vis-à-vis the European Commission
7. Restrictions to Marie Skłodowska-Curie Actions Individual Fellowship applications
8. The Swiss budget being excluded from the indicative budget lines leads to misunderstanding by project partners to exceed the European Union’s recommended budget ceiling
9. Increasing anti-Swiss-attitude in the European research landscape

At PGS Unit level, especially points 5 (added workload) and 6 (lack of coordinator role) were emphasized. In addition, EDCTP participation was felt challenged by both the PGS Unit and project leaders, owing to information websites discouraging project development or project contributions from Swiss institutions.

The lack of coordinator role led to a major challenge in the Swiss TPH funding strategy. In the EU framework programmes 5 to 7, co-investigator was the default role for Swiss TPH researchers in consortia. In 2013 a strategic decision by the directorate was taken to start acquiring projects in the coordinating role in the new Horizon 2020 programme as well as in EDCTP projects. Such initiation of consortia leads at Swiss TPH was entirely defeated by the political turbulences occurring in 2014 as demonstrated by a complete absence of consortia coordinator roles for collaborative project grants during 2013–2017. Consequently, this also meant that no funding was accessed for project management during these years, which is usually on the coordinators budget, and the intended increased visibility of Swiss TPH in the research community and towards the EC remained unchanged.

Many of those challenges were confirmed in a second, more systematic assessment addressing how the partial association of Switzerland affected Swiss TPH conducted in 2019, three years after the re-instatement of the associated status. For this, in a first arm of the survey, the main Swiss TPH applicants of each funding request to Horizon 2020 between 2014 and 2018 were consulted. Of 51 requests, 32 responses were received mostly via survey (face-to-face in two cases). Of the 32 respondents, 34.4% stated that their co-applicant had raised issues pertaining to Switzerland’s partial association during 2014–2016. Of 11 participants that specified the type of concern, 64% stated financial/budget issues, 18% stated an added administrative burden and 27% stated insecurities about the status of Switzerland. However, the large majority of participants (94%) felt that their external collaborators’ willingness or ability to collaborate was not affected by Switzerland’s third-party association status, neither for that given proposal nor in general. The majority of the respondents also suggested that Swiss TPH’s role in the Horizon 2020 proposal or consortium was not affected (88%) in the long-term and that their collaborators did not take advantage of Swiss TPH’s limiting situation during the grant development stage of the project (91%). On process-level, the majority of participants responded that the partial association of Switzerland did not cause any additional work before, during or after the proposal development (only 6.5% of participants stated to have experienced added work after proposal development) and that there were no financial or practical limitations to their contribution to the project that would not have arisen under full-association circumstances (for 12.5%, the partial association status did so). All except one respondent referred to a need for clarifications of status and/or budget and informing the members of the consortium; in this context, three of them explicitly mentioned exchange with or support by the PGS Unit. Three of five respondents expressed a continuous uncertainty about the status of Switzerland to be the main source of the additional time investment. In contrast, three respondents felt that the being excluded from the budget line gave Swiss institutions an advantage because this was (mistakenly) perceived as an addition to the overall budget.

To the question of whether they were ever rejected or excluded from collaborating in any grant proposal and project owing to Switzerland’s partial association, 12.5% stated yes, elaborating further that they were told by colleagues that they were reluctant to involve a Swiss institution or that they learned retrospectively from former partners/potential collaborators that they had submitted a proposal where Swiss TPH was not considered because of the status uncertainty. One fifth (21.9%) of the respondents felt that the partial association of Switzerland caused them personal limitations in (i) choosing a Horizon 2020 funding scheme that they would have applied to under normal circumstances; (ii) a preferred role within a consortium/collaboration or (iii) the choice of collaborations. Three participants stated that they were approached to act as partner in a proposal to EDCTP but had to reject owing to their ineligibility. However, the main problem was stated to be a drop in opportunities to act as co-investigator, which is the more frequent role in funding applications involving Swiss TPH. Project partners were hesitant to approach Swiss researchers owing to persistent uncertainty, fear of imbalance and less chance of getting the project funded. It was stated that association insecurities are still lingering and toxic. From the discussions in 2014 as well as the interviews in 2019 it became clear that that the exclusion from European research networks and the lack of consultation of expertise by the European partner institutions was seen as almost more harmful in the short- and long-term than the financial aspects of limited participation in EU funding schemes.

Comparing the two in-house assessments that were done, it is striking that the perceptions captured in the 2019 survey are more positive or neutral compared with the informal in-house assessment between PGS Unit and project leaders in 2014. Although many of the consequences raised (Table 1) were mentioned again in the 2019 survey by individual researchers, the majority seemed to perceive the past and current situation not as gloomy. In 2014, it was for instance pointed out that partners were less interested in cooperating with Swiss TPH and that Swiss TPH would therefore be less involved in funding proposals. In addition, an anti-Swiss attitude in the EU research community was noted. In the 2019 survey, however, almost all respondents (94%) felt that their external collaborators’ willingness or ability to collaborate was not affected by Switzerland’s partial association. This discrepancy in perception may suggest (i) that the issues summarized in 2014 were made out of anticipation and worry rather than actual experience, given the short exposure to this new situation at the time; (ii) that there was a recall bias owing to the three years passed since the end of the partial association period and the perception of gravity of events has somewhat faded [18]; and/or (iii) that the issues related to this difficult period have not lingered much, after all.

In the 2014 assessment, it was also stated that there were uncertainties, rumours and misunderstandings by coordinating partners toward Swiss eligibility and that more time had to be invested for proposal development and communication on the status of Switzerland. These points were also mentioned by the respondents in 2019 (external and internal); yet, the general perception was that the situation did not add a substantial amount of work to the grant planning, writing and execution process. The discrepancy of perception points towards a successful intervention by the PGS Unit, supported by the in-house finance team, in this exceptional scenario. They acted as information source internally and externally, as liaison body with both the EC and the SERI to keep up with developments and changes in rules pertaining to the association of Switzerland and as workforce compensating for the additional workload. By doing so, the in-house supporting units seem to have buffered the effects of the partial association assuring smooth procedures for the applying researchers in-house and their external partners.

In a second arm of the survey targeting the outside perspective of Swiss TPH’s impairment, a total of 37 non-Swiss collaborators that had taken part in a Horizon 2020 grant proposal with Swiss TPH between 2016 and 2019 were sent an online survey of which six participated (four co-investigators and two project coordinators). Only one participant expressed concerns about financial aspects elaborating that the role of Switzerland and the allowable overall budget and available budget caused major concerns at the time. Five participants stated that Switzerland’s third-party status did not concern their willingness or ability to collaborate with Swiss TPH. All participants suggested that Switzerland’s third-party status did not affect the role of Swiss TPH in the proposal or consortium. In general, the respondents did not think that the third-party status caused any additional work for them before, during or after the proposal development; neither did they experience any financial or practical limitation to the project that one would not have encountered under full-association circumstances. It is, however, noteworthy that only external respondents that continued their collaboration with Swiss TPH were included in the survey of which only 16.2% responded, providing a bias of the most devoted project partners (see limitations).

### Resilience of research and academic institutions in face of restricted access to national and regional funding

We identified the main lessons learned, from this institutional case study, that may be applicable to research and academic institutions across the globe and that are facing restrictions to regional or national funding because of geopolitical or other reasons that are changing the priorities of funders temporarily (e.g. Covid-19) or permanently (e.g. Brexit, climate change).

Firstly, while drawing from the qualitative and quantitative outcomes, we have also looked back at almost 80 years of Swiss TPH history and experience having grown from less than 100 staff in the 1990s to 800 staff today and having increased its annual income by more than a factor 10 in the same period. This growth together with an accumulation of world-leading expertise in tropical medicine, parasitology and global health would not have taken place to the same extent if it had not been for the decade-long partnerships with institutions in Côte d’Ivoire, Tanzania, the Lao People's Democratic Republic and elsewhere. While personal relations helped being included in opportunities during the Swiss partial association to Horizon 2020, institutional partnerships based on fairness and equality [19, 20] allowed for mutual growth and have proven to be essential supporting systems for us and our partners during severe disruption in the funding or the actual conduct of research [21].

Another reason for the resilience of Swiss TPH is a high diversification of funding sources. On the research level, the institute relies only moderately on EU funding owing to the domain and geographical focal areas outside Europe having therefore built up a portfolio of national and international funders. Moreover, Swiss TPH operates by a three-pronged approach hosting research, education and services activities. This allows for versatility and flexibility with regard to internal resource management and spectrum/choice of international partners and funders—the latter including funders of research, multilateral donors, banks and private sector companies. In face of the high degree of economic and geopolitical insecurity currently imposed by the Covid-19 pandemic, this business model has shown viable again, contrasting the experience of many institutes of higher education in English speaking high-income countries that are heavily reliant one revenues from foreign students [22, 23].

Finally, a fully functional grant support unit has shown to be absolutely essential. While this may be a given in many universities in high-income countries, the same generalization cannot be made for academic and research institutions in LMICs, putting them at an extreme disadvantage when facing an adverse national or regional funding climate. Successful strategies for increased resilience whilst experiencing funding fluctuations are summarized in Table 2.

**Table 2:** Institutional strategies that have been found successful in increasing resilience towards fluctuating national and regional research funding

On operational level
Availability of a grants support office that takes on the following added duties Is involved with the national and regional funders and act as conversation link between funding/government authorities and the institution Invests the necessary time for communication on the topic of eligibility status to partners Supports proposal development with partners for budget forms, legal statements and other added administrative burdens
On strategic level A three-pronged strategy of research, education and services allows for high flexibility in managing staff and a broader range of potential funders and partners Diversification of funding sources including a large proportion of non-regional and multilateral funders Pursuit of long-term partnerships built on mutual support on the levels of funding, capacity building and mutual shaping of research strategy

### Study limitations

The grant application data retrieved from Swiss TPH for FP7, Horizon 2020 and EDCTP is too limited to conclusively quantify effects of the part-time association of Switzerland to Swiss TPH grants numbers, success rate and funding obtained. While the FP7 and Horizon 2020 data was double-checked and completed with the EU CORDIS database, there is no such tool for EDCTP, relying on information supplied by the applicants of a given call. We can therefore not exclude that the quantitative information pertaining to EDCTP may be incomplete.

Due to the time passed between the period of partial association status of Switzerland a recall bias in answering the in-house survey may make negative effects likely to be underestimated.

Two types of limitations apply to the survey of the external collaborators; first, the number of respondents was very small (*n* = 6; 16%). Second, the external partners were identified by the in-house respondents naming their collaborators in their most recent Horizon 2020 project. Including only Horizon 2020 collaborators, it is likely that their responses are biased in favour of collaborating with Swiss institutions. However, it was beyond the scope of this work to try to identify and approach external respondents that consciously excluded collaborating Swiss TPH for a Horizon 2020 grant. Hence, the majority of missed out opportunities and lost long-term collaborations are not being reflected in this assessment.

One final limitation is that the authors that have conducted and written up this study are affiliated with the PGS Unit at Swiss TPH. A bias in interpreting the outcomes and observations made can therefore not be ruled out entirely.

### Outlook and conclusion

Switzerland finds itself yet again at a crossroad in EU–Swiss negotiations awaiting the outcomes of an internal consultation over an institutional framework agreement between the two parties that would consolidate mutual market access and set up the future relation. Meanwhile, the research community braces itself against any impact that may arise from a failure to agree on this framework. A potential degradation of relations at this point may result in limited access to ‘Horizon Europe’ that will be running from 2021 to 2027 with an overall budget of 100 billion Euro. This time, however, the exit of the UK from the EU (Brexit) as per 31st January 2020, adds a further complication to the Swiss cause. Like Switzerland, the UK will have to negotiate its association to Horizon Europe with the risk of the EC moving Switzerland to a lower priority position in any bilateral negotiations.

While the Swiss research landscape was considerably affected, the current analysis does not show any clear effects of Switzerland’s partial association to Horizon 2020 at the level of Swiss TPH. Immediate mitigation measures as described here were taken and are likely to have prevented more dramatic and visible effects. However, we would like to stress that, in the long-term, a perpetuating situation of insecurity and isolation from the global research community would be highly damaging for any research institution including ours. Alternative sources of funding can compensate for financial losses but not for the lack of trust by external partner organizations, the lack of pull for global talent and the loss of belonging to the European and, indeed, global research community. For Swiss TPH, rapprochement with the EU is the only way forward to ensure a research environment that is stable and trusted and that excels as part of the global research community. 

## Data Availability

All data generated or analysed during this study are included in the published article.
